# Dichlorido(ethanol-κ*O*)[2-(1,3-thia­zol-4-yl-κ*N*)-1*H*-benzimidazole-κ*N* ^3^]copper(II)

**DOI:** 10.1107/S1600536812013037

**Published:** 2012-03-31

**Authors:** Long Li, Kai-Sheng Diao, Yu-Qiu Ding, Jin-Niu Tang, Dai-Yin Wang

**Affiliations:** a College of Chemistry and Chemical Engineering, Guangxi University for Nationalities, Nanning 530006, People’s Republic of China; b Key Laboratory of Development & Application of Forest Chemicals of Guangxi, Nanning 530006, People’s Republic of China

## Abstract

In the title complex, [CuCl_2_(C_10_H_7_N_3_S)(C_2_H_5_OH)], the Cu^II^ ion is five-coordinated in a distorted square-pyramidal geometry by two N atoms from a 2-(1,3-thia­zol-4-yl)-1*H*-benzimidazole ligand, one O atom from an ethanol mol­ecule and two Cl atoms. In the crystal, O—H⋯Cl and N—H⋯Cl hydrogen bonds link the complex mol­ecules into a layer parallel to (100). π–π inter­actions between the thia­zole rings are observed [centroid–centroid distance = 3.749 (3) Å].

## Related literature

For related thia­bendazole complexes, see: Devereux *et al.* (2007 ▶); Umadevi *et al.* (1995 ▶).
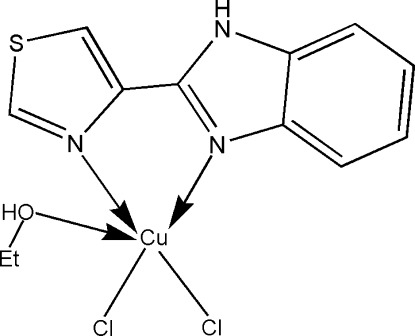


## Experimental

### 

#### Crystal data

[CuCl_2_(C_10_H_7_N_3_S)(C_2_H_6_O)]
*M* *_r_* = 381.75
Monoclinic, 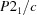

*a* = 13.928 (5) Å
*b* = 7.473 (3) Å
*c* = 16.653 (4) Å
β = 122.43 (2)°
*V* = 1463.0 (9) Å^3^
*Z* = 4
Mo *K*α radiation
μ = 2.00 mm^−1^
*T* = 296 K
0.35 × 0.33 × 0.32 mm

#### Data collection

Bruker APEX CCD diffractometer
Absorption correction: multi-scan (*SADABS*; Sheldrick, 1996 ▶) *T*_min_ = 0.542, *T*_max_ = 0.567
7540 measured reflections
2563 independent reflections
2139 reflections with *I* > 2σ(*I*)
*R*_int_ = 0.036

#### Refinement

*R*[*F* ^2^ > 2σ(*F* ^2^)] = 0.034
*wR*(*F* ^2^) = 0.109
*S* = 1.12
2563 reflections
182 parameters
1 restraint
H-atom parameters constrained
Δρ_max_ = 0.58 e Å^−3^
Δρ_min_ = −0.32 e Å^−3^

### 

Data collection: *SMART* (Bruker, 2007 ▶); cell refinement: *SAINT* (Bruker, 2007 ▶); data reduction: *SAINT* (Bruker, 2007 ▶); program(s) used to solve structure: *SHELXS97* (Sheldrick, 2008 ▶); program(s) used to refine structure: *SHELXL97* (Sheldrick, 2008 ▶); molecular graphics: *SHELXTL* (Sheldrick, 2008 ▶); software used to prepare material for publication: *SHELXTL* (Sheldrick, 2008 ▶).

## Supplementary Material

Crystal structure: contains datablock(s) I, global. DOI: 10.1107/S1600536812013037/hy2524sup1.cif

Structure factors: contains datablock(s) I. DOI: 10.1107/S1600536812013037/hy2524Isup2.hkl

Additional supplementary materials:  crystallographic information; 3D view; checkCIF report

## Figures and Tables

**Table 1:** Selected bond lengths (Å)

Cu1—N1	2.030 (3)
Cu1—N2	2.033 (3)
Cu1—Cl1	2.3194 (12)
Cu1—Cl2	2.2328 (12)
Cu1—O1	2.370 (3)

**Table 2:** Hydrogen-bond geometry (Å, °)

*D*—H⋯*A*	*D*—H	H⋯*A*	*D*⋯*A*	*D*—H⋯*A*
O1—H14⋯Cl1^i^	0.82	2.60	3.246 (3)	136
N3—H13⋯Cl1^ii^	0.86	2.59	3.431 (4)	165

Symmetry codes: (i) 
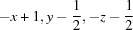
; (ii) 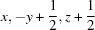.

**Fig. 1.**
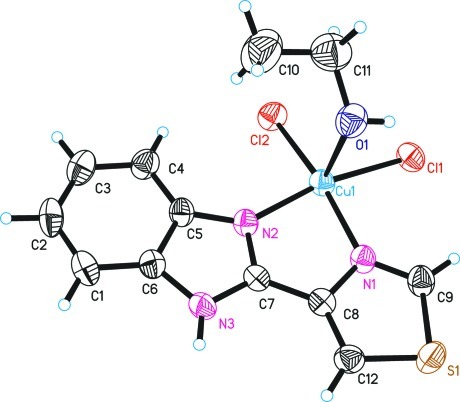
The molecular structure of the title compound. Displacement ellipsoids are drawn at the 50% probability level.

**Fig. 2.**
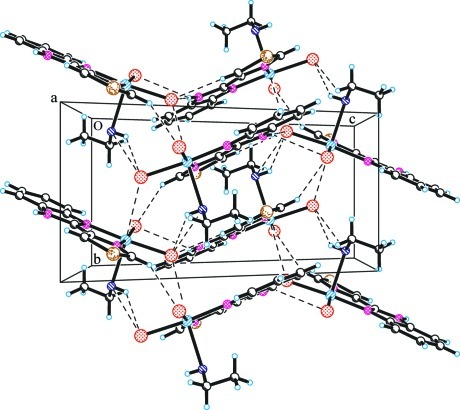
The crystal packing diagram of the title compound.

## supplementary crystallographic information

### Comment

As we know, thiabendazole, 2-(4-thiazolyl)benzimidazole, is widely used as a kind of anthelmintic. However, the insolubility in water restrict its potential efficacy. Thiabendazole has three N and one S atoms, easy to coordinate with non-toxic metals (Devereux *et al.*, 2007; Umadevi *et al.*, 1995). These metal-organic compounds would be more water soluble, yet retain the biological activity of the base. As part of our studies of researching the properties and effects of metal complexes of thiabendazole, we have synthesized the title compound.

In the title complex (Fig. 1), the Cu^II^ ion is five-coordinated in a distorted square-pyramidal geometry by two N atoms from a 1*H*-2-(4-thiazol-2-yl)benzimidazole ligand, one O atom from an ethanol molecule and two Cl atoms (Table 1). The dihedral angle between the imidazole ring (C5, C6, C7, N2, N3) and the thiazole ring (N1, S1, C8, C9, C12) is 3.8 (1)°. O—H···Cl and N—H···Cl hydrogen bonds link the complex molecules into a layer parallel to (100) (Fig. 2, Table 2). π–π interactions between the thiazole rings are observed [centroid–centroid distance = 3.749 (3) Å].

### Experimental

The title compound was prepared by the reaction of thiabendazole (1.5 mol) with cupric chloride (1 mol) in ethanol, with stirring at 343 K for 5 h and then filtered. The filtrate was kept at room temperature and three days later X-ray quality blue block-shaped single crystals were obtained.

### Refinement

H atoms were positioned geometrically and refined as riding atoms, with C—H = 0.93 (aromatic), 0.97 (methylene), 0.96 (methyl) and N—H = 0.86 Å and with *U*_iso_(H) = 1.2(1.5 for methyl)*U*_eq_(C, N). H atom of hydroxyl group was found from a difference Fourier map and refined as riding, with O—H = 0.82 Å and *U*_iso_(H) = 1.5*U*_eq_(O).

### Crystal data

**Table:**

[CuCl_2_(C_10_H_7_N_3_S)(C_2_H_6_O)]	*F*(000) = 772
*M**_r_* = 381.75	*D*_x_ = 1.733 Mg m^−^^3^
Monoclinic, *P*2_1_/*c*	Mo *K*α radiation, λ = 0.71073 Å
Hall symbol: -P 2ybc	Cell parameters from 2962 reflections
*a* = 13.928 (5) Å	θ = 2.5–28.0°
*b* = 7.473 (3) Å	µ = 2.00 mm^−^^1^
*c* = 16.653 (4) Å	*T* = 296 K
β = 122.43 (2)°	Block, blue
*V* = 1463.0 (9) Å^3^	0.35 × 0.33 × 0.32 mm
*Z* = 4	

### Data collection

**Table:**

Bruker APEX CCD diffractometer	2563 independent reflections
Radiation source: fine-focus sealed tube	2139 reflections with *I* > 2σ(*I*)
Graphite monochromator	*R*_int_ = 0.036
φ and ω scans	θ_max_ = 25.0°, θ_min_ = 1.7°
Absorption correction: multi-scan (*SADABS*; Sheldrick, 1996)	*h* = −16→16
*T*_min_ = 0.542, *T*_max_ = 0.567	*k* = −8→8
7540 measured reflections	*l* = −19→17

### Refinement

**Table:**

Refinement on *F*^2^	Primary atom site location: structure-invariant direct methods
Least-squares matrix: full	Secondary atom site location: difference Fourier map
*R*[*F*^2^ > 2σ(*F*^2^)] = 0.034	Hydrogen site location: inferred from neighbouring sites
*wR*(*F*^2^) = 0.109	H-atom parameters constrained
*S* = 1.12	*w* = 1/[σ^2^(*F*_o_^2^) + (0.0577*P*)^2^ + 0.818*P*] where *P* = (*F*_o_^2^ + 2*F*_c_^2^)/3
2563 reflections	(Δ/σ)_max_ = 0.001
182 parameters	Δρ_max_ = 0.58 e Å^−^^3^
1 restraint	Δρ_min_ = −0.32 e Å^−^^3^

### Special details

**Table:**

Geometry. All e.s.d.'s (except the e.s.d. in the dihedral angle between two l.s. planes) are estimated using the full covariance matrix. The cell e.s.d.'s are taken into account individually in the estimation of e.s.d.'s in distances, angles and torsion angles; correlations between e.s.d.'s in cell parameters are only used when they are defined by crystal symmetry. An approximate (isotropic) treatment of cell e.s.d.'s is used for estimating e.s.d.'s involving l.s. planes.
Refinement. Refinement of *F*^2^ against ALL reflections. The weighted *R*-factor *wR* and goodness of fit *S* are based on *F*^2^, conventional *R*-factors *R* are based on *F*, with *F* set to zero for negative *F*^2^. The threshold expression of *F*^2^ > σ(*F*^2^) is used only for calculating *R*-factors(gt) *etc*. and is not relevant to the choice of reflections for refinement. *R*-factors based on *F*^2^ are statistically about twice as large as those based on *F*, and *R*- factors based on ALL data will be even larger.

### Fractional atomic coordinates and isotropic or equivalent isotropic displacement parameters (Å^2^)

**Table:**

	*x*	*y*	*z*	*U*_iso_*/*U*_eq_	
Cu1	0.63072 (4)	0.19084 (6)	−0.14281 (3)	0.03262 (17)	
Cl1	0.51582 (8)	0.09392 (13)	−0.29888 (6)	0.0417 (3)	
Cl2	0.76893 (8)	0.27324 (14)	−0.16501 (7)	0.0424 (3)	
S1	0.31736 (8)	0.12748 (15)	−0.12506 (7)	0.0451 (3)	
O1	0.6837 (2)	−0.1091 (4)	−0.0919 (2)	0.0475 (7)	
H14	0.6287	−0.1424	−0.1428	0.071*	
N1	0.4997 (2)	0.1642 (4)	−0.1231 (2)	0.0323 (7)	
N2	0.7045 (2)	0.2934 (4)	−0.0093 (2)	0.0305 (6)	
N3	0.6867 (2)	0.3619 (4)	0.1119 (2)	0.0347 (7)	
H13	0.6561	0.3739	0.1448	0.042*	
C1	0.8865 (3)	0.4787 (5)	0.2244 (3)	0.0438 (9)	
H1	0.8786	0.5081	0.2749	0.053*	
C2	0.9871 (3)	0.5050 (5)	0.2291 (3)	0.0467 (10)	
H2	1.0488	0.5528	0.2844	0.056*	
C3	0.9993 (3)	0.4623 (6)	0.1540 (3)	0.0475 (10)	
H3	1.0686	0.4837	0.1600	0.057*	
C4	0.9115 (3)	0.3892 (6)	0.0708 (3)	0.0418 (9)	
H4	0.9206	0.3601	0.0211	0.050*	
C5	0.8079 (3)	0.3602 (5)	0.0638 (2)	0.0333 (8)	
C6	0.7975 (3)	0.4063 (5)	0.1408 (2)	0.0342 (8)	
C7	0.6359 (3)	0.2965 (4)	0.0229 (2)	0.0296 (7)	
C8	0.5201 (3)	0.2321 (4)	−0.0382 (2)	0.0302 (7)	
C9	0.3960 (3)	0.1024 (5)	−0.1753 (3)	0.0397 (9)	
H9	0.3675	0.0496	−0.2344	0.048*	
C10	0.8827 (4)	−0.1291 (9)	0.0142 (4)	0.0862 (18)	
H10A	0.8844	−0.0006	0.0146	0.129*	
H10B	0.9524	−0.1745	0.0235	0.129*	
H10C	0.8741	−0.1709	0.0645	0.129*	
C11	0.7867 (4)	−0.1919 (7)	−0.0774 (4)	0.0655 (13)	
H11A	0.7975	−0.1583	−0.1283	0.079*	
H11B	0.7809	−0.3212	−0.0770	0.079*	
C12	0.4314 (3)	0.2252 (5)	−0.0274 (3)	0.0384 (9)	
H12	0.4317	0.2673	0.0253	0.046*	

### Atomic displacement parameters (Å^2^)

**Table:**

	*U*^11^	*U*^22^	*U*^33^	*U*^12^	*U*^13^	*U*^23^
Cu1	0.0366 (3)	0.0378 (3)	0.0267 (3)	−0.00281 (18)	0.0191 (2)	−0.00050 (18)
Cl1	0.0483 (6)	0.0476 (6)	0.0280 (5)	−0.0005 (4)	0.0197 (4)	−0.0028 (4)
Cl2	0.0430 (5)	0.0529 (6)	0.0393 (5)	−0.0029 (4)	0.0274 (5)	0.0034 (4)
S1	0.0340 (5)	0.0540 (6)	0.0464 (6)	−0.0062 (4)	0.0211 (5)	−0.0006 (5)
O1	0.0417 (15)	0.0473 (17)	0.0467 (17)	0.0008 (12)	0.0193 (14)	0.0040 (13)
N1	0.0334 (16)	0.0382 (17)	0.0231 (15)	−0.0026 (13)	0.0137 (13)	0.0001 (12)
N2	0.0326 (15)	0.0330 (16)	0.0249 (15)	−0.0019 (12)	0.0148 (13)	0.0014 (12)
N3	0.0389 (17)	0.0408 (17)	0.0282 (16)	−0.0026 (13)	0.0205 (14)	−0.0046 (13)
C1	0.054 (2)	0.036 (2)	0.036 (2)	−0.0076 (18)	0.0208 (19)	−0.0076 (17)
C2	0.045 (2)	0.037 (2)	0.039 (2)	−0.0134 (17)	0.0101 (19)	−0.0046 (17)
C3	0.036 (2)	0.048 (2)	0.049 (2)	−0.0072 (18)	0.0173 (19)	0.005 (2)
C4	0.036 (2)	0.053 (2)	0.038 (2)	−0.0038 (17)	0.0209 (18)	0.0017 (18)
C5	0.0335 (19)	0.0355 (19)	0.027 (2)	−0.0027 (15)	0.0140 (16)	0.0004 (15)
C6	0.0348 (19)	0.0333 (19)	0.031 (2)	−0.0009 (15)	0.0156 (16)	−0.0006 (15)
C7	0.0353 (19)	0.0267 (18)	0.0276 (19)	0.0005 (14)	0.0173 (16)	0.0008 (14)
C8	0.0369 (19)	0.0245 (17)	0.0303 (19)	0.0007 (14)	0.0188 (16)	0.0046 (14)
C9	0.042 (2)	0.044 (2)	0.032 (2)	−0.0075 (17)	0.0193 (18)	−0.0026 (17)
C10	0.056 (3)	0.086 (4)	0.088 (4)	0.008 (3)	0.019 (3)	0.006 (3)
C11	0.070 (3)	0.054 (3)	0.072 (4)	0.009 (2)	0.038 (3)	0.008 (2)
C12	0.042 (2)	0.040 (2)	0.038 (2)	−0.0013 (16)	0.0257 (19)	−0.0007 (17)

### Geometric parameters (Å, º)

**Table:**

Cu1—N1	2.030 (3)	C1—H1	0.9300
Cu1—N2	2.033 (3)	C2—C3	1.387 (6)
Cu1—Cl1	2.3194 (12)	C2—H2	0.9300
Cu1—Cl2	2.2328 (12)	C3—C4	1.377 (5)
Cu1—O1	2.370 (3)	C3—H3	0.9300
S1—C9	1.707 (4)	C4—C5	1.400 (5)
S1—C12	1.712 (4)	C4—H4	0.9300
O1—C11	1.459 (5)	C5—C6	1.408 (5)
O1—H14	0.8200	C7—C8	1.451 (5)
N1—C9	1.308 (5)	C8—C12	1.343 (5)
N1—C8	1.379 (5)	C9—H9	0.9300
N2—C7	1.324 (4)	C10—C11	1.466 (7)
N2—C5	1.388 (4)	C10—H10A	0.9600
N3—C7	1.346 (5)	C10—H10B	0.9600
N3—C6	1.389 (4)	C10—H10C	0.9600
N3—H13	0.8600	C11—H11A	0.9700
C1—C2	1.375 (6)	C11—H11B	0.9700
C1—C6	1.385 (5)	C12—H12	0.9300
			
N1—Cu1—N2	80.28 (12)	C3—C4—H4	121.1
N1—Cu1—Cl2	169.60 (9)	C5—C4—H4	121.1
N2—Cu1—Cl2	95.86 (9)	N2—C5—C4	131.8 (3)
N1—Cu1—Cl1	90.62 (9)	N2—C5—C6	108.8 (3)
N2—Cu1—Cl1	169.49 (9)	C4—C5—C6	119.4 (3)
Cl2—Cu1—Cl1	92.21 (4)	C1—C6—N3	132.1 (3)
N1—Cu1—O1	89.08 (10)	C1—C6—C5	122.5 (3)
N2—Cu1—O1	95.01 (11)	N3—C6—C5	105.4 (3)
Cl2—Cu1—O1	100.92 (7)	N2—C7—N3	112.7 (3)
Cl1—Cu1—O1	90.06 (8)	N2—C7—C8	118.8 (3)
C9—S1—C12	90.03 (18)	N3—C7—C8	128.5 (3)
C11—O1—Cu1	123.4 (3)	C12—C8—N1	115.1 (3)
C11—O1—H14	109.5	C12—C8—C7	132.4 (3)
Cu1—O1—H14	88.7	N1—C8—C7	112.5 (3)
C9—N1—C8	111.0 (3)	N1—C9—S1	114.0 (3)
C9—N1—Cu1	134.1 (3)	N1—C9—H9	123.0
C8—N1—Cu1	114.8 (2)	S1—C9—H9	123.0
C7—N2—C5	105.8 (3)	C11—C10—H10A	109.5
C7—N2—Cu1	113.5 (2)	C11—C10—H10B	109.5
C5—N2—Cu1	140.6 (2)	H10A—C10—H10B	109.5
C7—N3—C6	107.3 (3)	C11—C10—H10C	109.5
C7—N3—H13	126.3	H10A—C10—H10C	109.5
C6—N3—H13	126.4	H10B—C10—H10C	109.5
C2—C1—C6	116.6 (4)	O1—C11—C10	107.6 (4)
C2—C1—H1	121.7	O1—C11—H11A	110.2
C6—C1—H1	121.7	C10—C11—H11A	110.2
C1—C2—C3	122.0 (4)	O1—C11—H11B	110.2
C1—C2—H2	119.0	C10—C11—H11B	110.2
C3—C2—H2	119.0	H11A—C11—H11B	108.5
C4—C3—C2	121.7 (4)	C8—C12—S1	109.9 (3)
C4—C3—H3	119.1	C8—C12—H12	125.1
C2—C3—H3	119.1	S1—C12—H12	125.1
C3—C4—C5	117.7 (4)		
			
N1—Cu1—O1—C11	−174.0 (3)	C2—C1—C6—N3	179.7 (4)
N2—Cu1—O1—C11	−93.8 (3)	C2—C1—C6—C5	0.3 (6)
Cl2—Cu1—O1—C11	3.1 (3)	C7—N3—C6—C1	179.7 (4)
Cl1—Cu1—O1—C11	95.4 (3)	C7—N3—C6—C5	−0.8 (4)
N2—Cu1—N1—C9	179.9 (4)	N2—C5—C6—C1	−179.3 (3)
Cl2—Cu1—N1—C9	111.0 (5)	C4—C5—C6—C1	−0.5 (6)
Cl1—Cu1—N1—C9	5.2 (3)	N2—C5—C6—N3	1.1 (4)
O1—Cu1—N1—C9	−84.9 (4)	C4—C5—C6—N3	180.0 (3)
N2—Cu1—N1—C8	3.3 (2)	C5—N2—C7—N3	0.4 (4)
Cl2—Cu1—N1—C8	−65.6 (6)	Cu1—N2—C7—N3	178.9 (2)
Cl1—Cu1—N1—C8	−171.4 (2)	C5—N2—C7—C8	−179.4 (3)
O1—Cu1—N1—C8	98.5 (2)	Cu1—N2—C7—C8	−0.9 (4)
N1—Cu1—N2—C7	−1.2 (2)	C6—N3—C7—N2	0.3 (4)
Cl2—Cu1—N2—C7	169.0 (2)	C6—N3—C7—C8	−179.9 (3)
Cl1—Cu1—N2—C7	29.1 (6)	C9—N1—C8—C12	−1.4 (4)
O1—Cu1—N2—C7	−89.4 (2)	Cu1—N1—C8—C12	175.9 (2)
N1—Cu1—N2—C5	176.5 (4)	C9—N1—C8—C7	178.1 (3)
Cl2—Cu1—N2—C5	−13.2 (4)	Cu1—N1—C8—C7	−4.5 (4)
Cl1—Cu1—N2—C5	−153.1 (4)	N2—C7—C8—C12	−177.0 (4)
O1—Cu1—N2—C5	88.3 (4)	N3—C7—C8—C12	3.2 (6)
C6—C1—C2—C3	0.3 (6)	N2—C7—C8—N1	3.7 (4)
C1—C2—C3—C4	−0.8 (7)	N3—C7—C8—N1	−176.2 (3)
C2—C3—C4—C5	0.6 (6)	C8—N1—C9—S1	1.2 (4)
C7—N2—C5—C4	−179.6 (4)	Cu1—N1—C9—S1	−175.49 (19)
Cu1—N2—C5—C4	2.5 (7)	C12—S1—C9—N1	−0.6 (3)
C7—N2—C5—C6	−0.9 (4)	Cu1—O1—C11—C10	78.2 (5)
Cu1—N2—C5—C6	−178.8 (3)	N1—C8—C12—S1	1.0 (4)
C3—C4—C5—N2	178.6 (4)	C7—C8—C12—S1	−178.4 (3)
C3—C4—C5—C6	0.0 (5)	C9—S1—C12—C8	−0.3 (3)

### Hydrogen-bond geometry (Å, º)

**Table:**

*D*—H···*A*	*D*—H	H···*A*	*D*···*A*	*D*—H···*A*
O1—H14···Cl1^i^	0.82	2.60	3.246 (3)	136
N3—H13···Cl1^ii^	0.86	2.59	3.431 (4)	165

Symmetry codes: (i) −*x*+1, *y*−1/2, −*z*−1/2; (ii) *x*, −*y*+1/2, *z*+1/2.
